# The Epidemiology of Meningococcal Disease and Carriage, Genotypic Characteristics and Antibiotic Resistance of *Neisseria meningitidis* Isolates in Zhejiang Province, China, 2011–2021

**DOI:** 10.3389/fmicb.2021.801196

**Published:** 2022-01-24

**Authors:** Yunyi Zhang, Xuan Deng, Yan Jiang, Junyan Zhang, Li Zhan, Lingling Mei, Hangjing Lu, Pingping Yao, Hanqing He

**Affiliations:** ^1^Zhejiang Provincial Center for Disease Control and Prevention, Hangzhou, China; ^2^Department of Infectious Diseases, Sir Run Run Shaw Hospital, Zhejiang University School of Medicine, Hangzhou, China

**Keywords:** meningococcal disease, carriage, epidemiology, antibiotic resistance, *Neisseria meningitidis*, multilocus sequence typing (MLST)

## Abstract

*Neisseria meningitidis* (*Nm*) remains a worldwide leading cause of epidemic meningitis. During 2011–July 2021, 55 meningococcal disease (MD) cases were reported with a case fatality rate of 5.45% in Zhejiang Province, China. The median age was 7 years. The annual incidence was 0.0017–0.0183 per 100,000 population. The highest age-specific incidence was observed in the group younger than 1 year. Serogroup was identified in 30 laboratory-confirmed MD cases, and MenB was most predominant. MenB was mainly observed in two age groups: younger than 5 and older than 35 years. MenB incidence was significantly increasing from 0.0018 per 100,000 in 2013 to 0.0070 per 100,000 in 2019. During 2015–2020, 17 positive samples were detected from 2,827 throat swabs from healthy population, of which 70.59% was MenB. Twenty multilocus sequence typing sequence types (STs) containing eight newly assigned STs (ST15881–ST15888) were determined in all *Nm* isolates. Either in MD cases or in healthy population, MenB CC ST-4821 was the predominant ST. It was worth noting that two MenY CC ST-23 cases occurred in 2019 and 2021, respectively. MenY CC ST-23 MD cases increased gradually in China. Phylogeny results based on genome sequencing indicated that Chinese MenW CC ST-11 isolates were genetically linked and grouped together with Japanese isolates, separated from MenW CC ST-11 isolates from Saudi Arabia Hajj outbreak, Europe, South Africa, South America, North America, and Oceania. MenW CC ST-11 isolates from East Asia might have evolved locally. Antibiotic susceptibility tests revealed a relatively high resistance rate (22.86%) of *Nm* isolates to penicillin. This study provided valuable data for Chinese public health authorities to grasp the temporal epidemiological characteristics of MD and healthy carriage.

## Introduction

*Neisseria meningitidis* (*Nm*), an obligate human bacterial pathogen, can cause severe, life-threatening meningococcal disease (MD) with case fatality rates (CFRs) of 10–20% even with prompt treatment (MacNeil et al., 2018), typically reported in infants younger than 1 year and adolescents/young adults (Borrow et al., 2017). Up to 20% of survivors suffer from long-term sequelae including deafness, loss of limbs, and seizure (Whittaker et al., 2017). Besides classical manifestation as meningitis or septicemia, few cases of bacteremic pneumonia, primary lumbar spondylitis, pericarditis, cellulitis, septic arthritis, and primary polyarthritis due to *Nm* infection have been reported worldwide, mostly in elder adults, children, or individuals with comorbidities (Yubini et al., 2018; Gomez et al., 2019).

The epidemiology of MD varies considerably across geographical locations, time periods, and age of the hosts (Pelton, 2016; Borrow et al., 2017). In recent years, most regions of the world have experienced a downward trend in the incidence of MD (Li et al., 2018). In China, the incidence has declined sharply from the peak of 403 cases per 100,000 population in 1967 to 0.047 cases per 100,000 population from 2006 to 2014 (Li et al., 2015). However, the current overall incidence rate of China might be underestimated according to subnational estimates of 1.84 cases per 100,000 population (Zhang et al., 2016).

Accompanying the decreased incidence, the prevalence of different serogroups has changed substantially globally. Control of MD caused by serogroup A has been achieved to a certain extent across the “meningitis belt” of Africa where mass immunization programs with meningococcal A conjugate vaccines (MenAfriVac) have been implemented since 2010 (Novak et al., 2019). Between 2010 and 2015, nine meningitis belt countries observed a 99% reduction in MenA disease incidence (Trotter et al., 2017). Meanwhile, MD caused by serogroups other than NmA carries a substantial disease burden in the sub-Saharan meningitis belt countries (Bozio et al., 2018; Fernandez et al., 2019). An epidemiological shift has happened from the predominance of a single serogroup to diverse causes of MD or epidemics. Successful introduction of vaccination against MenA, MenC, and, more recently, MenB, has controlled and prevented MD caused by associated serogroups effectively in European countries including the United Kingdom, the Asia Pacific region, and the African meningitis belt (Trotter et al., 2017; Li et al., 2018; Booy et al., 2019; Aye et al., 2020; Ladhani et al., 2020). However, unexpected surges in the prevalence of certain serogroups occasionally occurred, for example, MenY caused epidemics in Scandinavia between 2010 and 2012 (Broker et al., 2014), serogroup predominance switch from MenB to MenW in Chile in 2012 (Villena et al., 2019). In China, serogroup A was continuously predominant from 1960s to 1980s, responsible for more than 95% of MD cases (Hu, 1991). With the implementation of MenA polysaccharide meningococcal vaccine since 1982, the incidence rate declined steadily (Liu et al., 2000; Deng et al., 2012). On the contrary, the proportion of MenB-caused sporadic cases rose apparently (Li et al., 2015). During the course of 2015–2017, 54.08% of clinical cases were due to MenB infection (Li J. H. et al., 2019). The dynamic and unpredictable epidemiology proposed a particular challenge for MD prevention (Booy et al., 2019). Ongoing surveillance is essential for both insight into temporal MD epidemiology and to provide fundamental data for vaccine formulation, vaccine policy, and monitoring the impact of implemented vaccines.

*Neisseria meningitidis* is a commensal inhabitant of the human upper respiratory tract. It colonizes 2–20% of healthy individuals in the population (Chamorro et al., 2019). Healthy humans colonized by meningococci have been considered to be the main source of *Nm* transmission (Yazdankhah and Caugant, 2004; MacLennan et al., 2006). Under certain conditions such as the causative serogroup, the host immune system, and some environmental factors, the development of MD from carriage may occur (Kim et al., 2017). Carriage studies of *Nm* for healthy population will be helpful to understand in depth the circulating *Nm* from MD cases and carriage and detect potential hyperinvasive isolates in healthy population. It is valuable for MD control and prevention. The aims of this study were (i) to put insight into the epidemiology of both MD and carriage in Zhejiang Province, China, from 2011 to 2021 and (ii) to reveal the genotypic characteristics and antibiotic resistance of *Nm* isolates.

## Materials and Methods

### Data Collection

Surveillance of MD is mandatory in China. MD cases are notified to the National Notifiable Diseases Surveillance System by physicians nationwide since 2005. Public health specialists from local Centers for Disease Control and Prevention conduct a face-to-face investigation to collect information on demographic and epidemiological characteristics, clinical manifestation, vaccination status, laboratory test, and disease outcome for each MD case. To meet provincial monitoring requirements, investigation on the carriage rate of *Nm* in healthy population of all ages through throat swab has been performed in Jiaojiang, Cangnan, Yiwu, Jingdong, Longquan, Yinzhou, and Haining, respectively, during 2015–2020. Normally, subjects were divided into 7- to 8-year age groups, each of which included 30–50 individuals.

### Case Definition

Confirmed case: detection of *Nm* nucleic acid by validated polymerase chain reaction (PCR) assay or isolation of *Nm* from specimen obtained from blood, cerebrospinal fluid (CSF), or skin lesion. Clinical case: present with typical symptoms without laboratory-confirmed results, such as fever, headache, vomiting, meningeal irritation, or purpura fulminans (Li et al., 2015). Associated sample collection and laboratory techniques were according to the National Surveillance Program for Meningococcal Meningitis (Ministry of Health of People’s Republic of China, 2006) and the National Diagnosis Standard for Meningococcal Meningitis (WS 295-2019, Ministry of Health of People’s Republic of China, 2019).

### Isolation, Identification, and Serotyping of *Neisseria meningitidis*

Blood, CSF, skin lesion, or swab samples, and so on, were directly plated onto the chocolate agar within 4 h of sample collection and incubated at 35°C in a 5% CO_2_-enriched atmosphere for 72 h. Presumptive *Neisseria* colonies with typical morphology were subcultured on blood agar enriched with 5% sheep blood and subjected to further identification by using VITEK 2 compact system with NH Cards (bioMérieux, Lyon, France). If necessary, 16S rDNA was amplified and sequenced for identification (Walcher et al., 2013). Confirmed *Nm* isolates were serogrouped by slide agglutination using commercial antisera according to the manufacturer’s instruction (Remel, United Kingdom) and PCR method (Taha, 2000; Claus et al., 2002; Bennett et al., 2004; Fraisier et al., 2009).

### Genome Sequencing and Assembly

The total DNA of *Nm* isolates was extracted by using Bacterial DNA Kit (Omega, United States). DNA purity quotient was tested by using spectrophotometer NanoDrop™ 2000 (Thermo Fisher Scientific, Waltham, MA, United States), and integrity was assessed by using electrophoresis on 0.8% agarose gel (Thermo Fisher Scientific, United States). Purified DNA with an amount greater than 50 ng was used for sequencing library preparation. DNA was simultaneously fragmented and tagged with adapters by using the TruePrep™ DNA Library Prep Kit V2 for Illumina (Vazyme, China). Individual library was assessed on the QIAxcel Advanced Automatic Nucleic Acid Analyzer and then quantified through qualitative PCR (qPCR) by using KAPA SYBR^®^ FAST qPCR Kits (Roche, Switzerland). The prepared library was sequenced on Illumina HiSeq X Ten platform (Illumina Inc., United States); 150-bp paired-end reads were generated. The reads were *de novo* assembled by using the software Unicycler. All draft genome sequences were submitted to PubMLST *Neisseria* database^1^.

### Molecular Typing Based on Genome Sequencing

The genome sequence of each *Nm* isolate was submitted to PubMLST *Neisseria* database (see text footnote 1) and aligned to multilocus sequence typing (MLST) housekeeping genes *abcZ*, *adk*, *aroE*, *fumC*, *gdh*, *pdhC*, and *pgm* alleles and variants of three Finetyping antigen coding sequences variable regions *porA*-VR1, *porA*-VR2, and *fetA*-VR on the bacterial isolate genome sequence database (BIGSdb) platform. MLST sequence type (ST), clonal complex (CC), and Finetyping antigens subtype were determined to each isolate based on alignment results. New MLST alleles, STs, and variants of Finetyping antigens (PorA and FetA) were designated by the *Neisseria* profile/sequence definitions database (see text footnote 1).

### Genome Comparisons

Three MenW CC ST-11 isolates (NM15, NM18, and NM19) were genome sequenced with N50 values of 63,116–75,833 and contig (≥200 bp) numbers of 121–129. Genomes of additional international MenW CC ST-11 strains used for comparisons were accessible in the *Neisseria* PubMLST database (see text footnote 1). A total of 483 isolates from MD outbreaks, epidemics, endemics, and sporadic cases across the world were involved (Supplementary Table 1). The PubMLST genome comparator tool was used to perform genome comparisons. Analysis type was against defined loci, which include 1,605 *Nm* core genes in *Nm* cgMLST v1.0 (Bratcher et al., 2014). Minimum identity and minimum alignment were set at 70 and 50%, respectively. When the distance matrix was calculated, incomplete loci (due to incomplete assembly) were ignored in pairwise comparison, loci paralogous in all were excluded, pairwise paralogous loci were excluded, and core threshold was 90%. The phylogenetic tree based on distance matrix was visualized by using SplitsTree4 version 4.13.1^2^.

### Antibiotic Susceptibility Tests

Antibiotic susceptibility of *Nm* isolates was assayed by using Etest strips (Antu, China). The minimum inhibitory concentrations (MICs) for each isolate to different antibiotics were measured after incubation at 35°C for 20–24 h in 5% CO_2_ on Mueller–Hinton agar with 5% sheep blood. Breakpoints for penicillin, ampicillin, azithromycin, rifampicin, minocycline, ciprofloxacin, ceftriaxone, trimethoprim-sulfamethoxazole, meropenem, levofloxacin, cefotaxime, and chloramphenicol were according to the Clinical and Laboratory Standards Institute (CLSI) documents M100 30th edition guidelines (Clinical and Laboratory Standards Institute [CLSI], 2020). *Nm* isolates resistant to three or more types of antibiotics belonging to different antibiotic classes were determined as multidrug-resistant (Magiorakos et al., 2012). *Escherichia coli* ATCC 25922 was used as a quality control strain.

### Statistical Analysis

Descriptive analysis was performed with SPSS 23.0. Graphs and tables were processed with Microsoft Excel 365. Correlation test for two quantitative variables was performed using the Pearson correlation test.

## Results and Discussion

### Epidemiology of Meningococcal Disease and Carriage in Zhejiang Province

During 2011–July 2021, 55 sporadic cases of MD were reported, 48 (87.27%) were laboratory confirmed, and 7 (12.73%) were clinically diagnosed. Overall, three cases were fatal, with an average CFR of 5.45%. The median age of those MD cases was 7 years, with the ages ranging from 12 days to 79 years old, with 31 cases aged 15 years (56.36%), occupying the highest percentage; 37 cases were male (67.27%).

During 2011–2020, the annual incidence was 0.0017–0.0183 per 100,000 population. The mortality rate was 0–0.0018 per 100,000 population. Year 2018 demonstrated the highest CFR of 25%. It was noteworthy that only one case of MD was detected in 2020, when the COVID-19 pandemic swept in China. We speculated that the strict mitigation measures against COVID-19 pandemic in China (Special Expert Group for Control of the Epidemic of Novel Coronavirus Pneumonia of the Chinese Preventive Medicine Association, 2020) might have caused a great impact on the spread and case detection of MD. For example, wearing of mask in public, limited scope of movements, and decreased willingness to move of the population could efficiently reduce the spread of respiratory infectious diseases. The highest age-specific incidence was observed in the age group of younger than 1 year (range, 0.0445–0.5013 per 100,000), followed by the age group of 1–4 years (range, 0–0.1065 per 100,000) and 10–19 years (range, 0–0.0524 per 100,000). Details are shown in Table 1.

**TABLE 1:** Cases of meningococcal disease by year, age, gender, and serogroup during 2011–July 2021 in Zhejiang Province, China.

Characteristic	Cases of year											
	2011	2012	2013	2014	2015	2016	2017	2018	2019	2020	July 2021	Total
	6 (0.0183)	10 (0.0183)	4 (0.0073)	6 (0.0109)	4 (0.0073)	4 (0.0072)	6 (0.0107)	4 (0.0071)	7 (0.0122)	1 (0.0017)	3 (0.0051)	55 (0.0089)

**Age group**												
<1 year	2 (0.4234)	1 (0.2273)	2 (0.4606)	1 (0.2222)	2 (0.4432)	1 (0.0445)	2 (0.3442)	2 (0.3429)	3 (0.5013)	1 (0.1576)	2 (0.3233)	19 (0.3230)
1–4 years	2 (0.1065)	1 (0.0508)	0 (0.0000)	1 (0.0503)	0 (0.0000)	2 (0.0205)	0 (0.0000)	2 (0.1023)	0 (0.0000)	0 (0.0000)	0 (0.0000)	8 (0.0356)
5–9 years	0 (0.0000)	0 (0.0000)	0 (0.0000)	1 (0.0349)	0 (0.0000)	0 (0.0000)	0 (0.0000)	0 (0.0000)	0 (0.0000)	0 (0.0000)	0 (0.0000)	1 (0.0034)
10–19 years	2 (0.0319)	3 (0.0524)	1 (0.0183)	1 (0.0190)	0 (0.0000)	0 (0.0000)	0 (0.0000)	0 (0.0000)	1 (0.0191)	0 (0.0000)	0 (0.0000)	8 (0.0138)
20–29 years	0 (0.0000)	1 (0.0106)	0 (0.0000)	2 (0.0213)	1 (0.0106)	1 (0.0022)	1 (0.0107)	0 (0.0000)	2 (0.0216)	0 (0.0000)	1 (0.0138)	9 (0.0094)
30–59 years	0 (0.0000)	4 (0.0150)	1 (0.0037)	0 (0.0000)	0 (0.0000)	0 (0.0000)	1 (0.0038)	0 (0.0000)	1 (0.0038)	0 (0.0000)	0 (0.0000)	7 (0.0023)
≥60 years	0 (0.0000)	0 (0.0000)	0 (0.0000)	0 (0.0000)	1 (0.0120)	0 (0.0000)	2 (0.0222)	0 (0.0000)	0 (0.0000)	0 (0.0000)	0 (0.0000)	3 (0.0029)

**Gender**												
Male	3 (0.0107)	8 (0.0286)	2 (0.0071)	5 (0.0177)	3 (0.0104)	3 (0.0106)	3 (0.0105)	3 (0.0104)	4 (0.0136)	0 (0.0000)	3 (0.0100)	37 (0.0117)
Female	3 (0.0113)	2 (0.0075)	2 (0.0075)	1 (0.0037)	1 (0.0038)	1 (0.0037)	3 (0.0110)	1 (0.0036)	3 (0.0107)	1 (0.0035)	0 (0.0000)	18 (0.0060)

**Serogroup**												
B	0 (0.0000)	0 (0.0000)	1 (0.0018)	0 (0.0000)	2 (0.0036)	1 (0.0018)	3 (0.0054)	3 (0.0053)	4 (0.0070)	0 (0.0000)	2 (0.0034)	16 (0.0026)
C	0 (0.0000)	1 (0.0019)	0 (0.0000)	1 (0.0018)	0 (0.0000)	1 (0.0018)	2 (0.0036)	0 (0.0000)	1 (0.0017)	0 (0.0000)	0 (0.0000)	6 (0.0010)
W	1 (0.0018)	0 (0.0000)	1 (0.0018)	2 (0.0036)	0 (0.0000)	0 (0.0000)	1 (0.0018)	0 (0.0000)	0 (0.0000)	0 (0.0000)	0 (0.0000)	5 (0.0008)
X	0 (0.0000)	0 (0.0000)	1 (0.0018)	0 (0.0000)	0 (0.0000)	0 (0.0000)	0 (0.0000)	0 (0.0000)	0 (0.0000)	0 (0.0000)	0 (0.0000)	1 (0.0002)
Y	0 (0.0000)	0 (0.0000)	0 (0.0000)	0 (0.0000)	0 (0.0000)	0 (0.0000)	0 (0.0000)	0 (0.0000)	1 (0.0017)	0 (0.0000)	1 (0.0017)	2 (0.0003)
Untyped/untypable	5 (0.0092)	5 (0.0092)	1 (0.0018)	1 (0.0018)	2 (0.0036)	1 (0.0018)	0 (0.0000)	1 (0.0018)	1 (0.0017)	1 (0.0017)	0 (0.0000)	18 (0.0029)

*Numbers in parentheses were the incidence per 100,000 population.*

During the study period, serogroup was identified in 30 laboratory-confirmed MD cases (62.50%). The remaining 18 confirmed cases were not identified. MenB was the most predominant (16 cases, 53.33%), followed by MenC [six cases (20%)] and MenW [five cases (16.7%)]. MenA had not been detected since 2011 in Zhejiang Province. MenW, MenX, and MenY were first isolated from MD cases in years 2011, 2013, and 2019, respectively, in Zhejiang Province. Since the late 1970s, MenB has spread and caused sporadic cases, even epidemics, worldwide. In 2010, MenB infection led to approximately 70% of MD cases in Europe (Trotter et al., 2007; Kriz et al., 2011; Whittaker et al., 2017). The high prevalence of MenB also occurred in the United States, where more than half of MD cases among individuals aged 16–20 years were attributed to MenB (MacNeil et al., 2018). Herein, 53.3% of MD cases were due to MenB, consistent with a previous report in China (Li J. H. et al., 2019), indicating that with the extensive implementation of MenA polysaccharide vaccine (MPV-A) since the 1980s, MenA plus MenC polysaccharide vaccine (MPV-AC) since 2003, and serogroup ACWY meningococcal polysaccharide vaccine (MPV-ACWY) since 2006 (Li et al., 2018), the predominant serogroup has changed gradually from MenA and MenC to MenB in the last few years in China. Given that a vaccine against MenB is still unavailable in China, this change of serogroup prevalence might pose the potential risk of epidemics of MenB.

**FIGURE 1:**
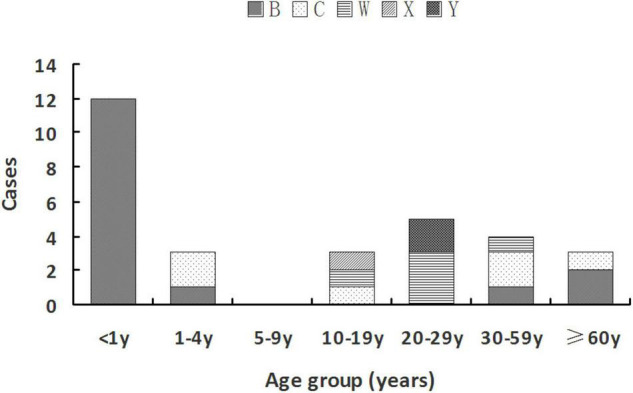
Serogroup distribution of *Nm* among different age groups during 2011–July 2021 in Zhejiang Province.

MenB was mainly observed in two age groups: younger than 5 years [13 cases (81.3%)] and older than 35 years [three cases (18.75%)]. MenC was found in age groups of 1–4 years [two cases (33.3%)], 10–19 years [one case (16.7%)], 30–59 years [two cases (33.3%)], and 60 years or older [one case (33.33%)]. MenW was detected in the middle age groups of 10–59 years. The only case with MenX was at the age of 18 years. Two MenY cases both belonged to the 20- to 29-year-old group (22.22%). Details are shown in Figure 1.

**FIGURE 2:**
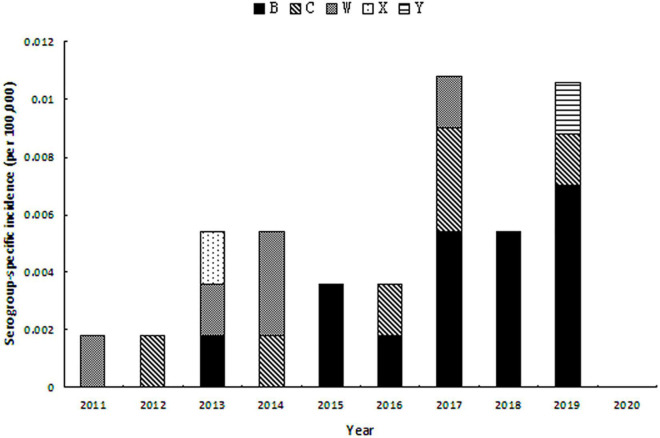
Serogroup-specific incidence of MD during 2011–2020 in Zhejiang Province.

As to the serogroup-specific incidence, during 2011–2020 (Figure 2), MenB incidence was significantly increasing from 0.0018 per 100,000 in 2013 to 0.0070 per 100,000 in 2019 (*r* = 0.906, *P* = 0.001). MenC incidence seemed to be stable when case was detected. MenY cases occurred in 2019 with the incidence rate of 0.0017 per 100,000.

Although Zhejiang Province had carried out the surveillance on carriage rate of *Nm* in healthy population since 2010, positive pathogen from throat cultures was first isolated in 2015. During 2015–2020, 17 positive culture isolates were detected from 2,827 throat swabs (0.64%) (Table 2). Analysis revealed that the distribution of serogroups showed high homogeneity in healthy population, 12 samples (70.59%) were MenB, and 1 sample (5.88%) each for MenC and MenY. MenB was the most predominant serogroup both in MD cases and in healthy population. In addition, three carriage isolates were identified as untypable, with a percentage of 17.65%. According to previously published reports, we found that the proportion of untypable *Nm* isolates from carriages was variable in different geographic regions and different populations. For example, the percentage of untypable *Nm* isolates from the 1- to 29-year-old population in Burkina Faso, one of the African meningitis belt countries, before the introduction of a meningococcal serogroup A conjugate vaccine was 13.3% (Kristiansen et al., 2011), which was close to that in our study. Meanwhile, in Paraguay, the proportion attained 31% in children and young adults (Chamorro et al., 2019). The majority of *Nm* isolates from healthy population in seven countries of the African meningitis belt (52%) (MenAfriCar Consortium, 2015) and Southern Ethiopia (76.4%) were untypable (Bårnes et al., 2016). So the proportion of untypable *Nm* isolates from carriage might be relevant to geographic regions and population.

**TABLE 2:** Surveillance on carriage rate of *Nm* in healthy population during 2015–2020.

Year	No. of subjects	No. of positive samples	Positive (%)	Serogroup (case)			
				B	C	Y	Untypable
2015	660	2	0.30	1	1	–	–
2016	915	2	0.22	1	–	–	1
2019	634	11	1.74	8	–	1	2
2020	618	2	0.32	2	–	–	–
Total	2,827	17	0.60	12	1	1	3

The median age of subjects with MenB was 23 years, and the range was from 9 months of age to 50 years old. Approximately 42% of subjects with MenB were aged 30–59 years, and 33% of those were aged 5–9 years. Subject with MenC was a 7-year-old child in 2016. Subject with MenY was a 23-year-old policeman.

### Multilocus Sequence Typing and Finetyping Antigen Analysis

Thirty-five *Nm* isolates from both MD cases and healthy population were further molecular typed (Table 3). Twenty different STs were classified, among which eight were designated new STs from ST15881 to ST15888. STs were assigned to six CCs and seven singletons. The Simpson index (DI) was 0.9317. In the healthy population, 38.46% (5/13) of the isolates belonged to MenB CC ST-4821. MenB CC ST-4821 was predominant in either MD cases with the proportion of 54.55% (12/22) or in carriers. Two MenC CC ST-4821 isolates were detected in two sporadic cases. Since 1978 when MenB and MenC CC ST-4821 strains were originally isolated (Yang et al., 2008), CC ST-4821 was mainly reported in China and associated with healthy carriers. In 2003, an outbreak caused by MenC CC ST-4821 was first reported in Anhui Province of China. In subsequent years, MenC CC ST-4821 became one of the leading CCs across China (Zhou et al., 2012). MenB CC4821 was also dominant among serogroup B strains. Different from MenC CC ST-4821, MenB CC4821 was usually associated with sporadic infections (Zhou et al., 2012). A MenX ST7 (CC ST-5) strain was isolated from an MD case in Wenzhou in 2013. Our previous studies (Pan et al., 2014; Ji et al., 2017) have determined that this strain arose from capsule switching between a MenX and a MenA ST-7 strain. *Nm* is naturally competent for exogenous DNA uptake and horizontal exchange in all growth phases, which enables gene exchange at the capsule polysaccharide biosynthesis locus between different strains resulting in capsule switching.

**TABLE 3:** MLST and Finetyping antigen characteristics of *Nm* isolates from both MD cases and carriage.

Isolates	Year	Source	Serogroup	abcZ	adk	aroE	fumC	gdh	pdhC	pgm	ST	CC	porA-VR1	porA-VR2	*fetA*-VR	Finetyping antigens
**MD cases isolates**																
Nm01	2011	Blood	W	2	3	4	3	8	4	6	11	ST-11	5	2	F1-1	P1.5, 2: F1-1
Nm02	2012	Blood	C	222	3	58	275	30	5	255	4,821	ST-4821	7-2	14	F3-3	P1.7-2, 14: F3-3
Nm03	2013	Blood	X	1	1	2	1	3	2	19	7	ST-5	20	9	F3-1	P1.20, 9: F3-1
Nm04	2013	CSF	W	2	3	4	3	8	4	6	11	ST-11	5	2	F1-1	P1.5, 2: F1-1
Nm05	2014	CSF	W	2	3	4	3	8	4	6	11	ST-11	5	2	F1-1	P1.5, 2: F1-1
Nm06	2014	Blood	B	6	2	53	9	259	25	20	15,885[Table-fn t1fns1]	–	22	14	F5-46	P1.22, 14: F5-46
Nm07	2015	CSF	B	222	3	58	261	263	5	255	3,200	ST-4821	20	14	F3-9	P1.20, 14: F3-9
Nm08	2015	CSF	B	222	3	58	1	386	18	255	10,051	ST-4821	20	23-7	F1-91	P1.20, 23-7: F1-91
Nm09	2017	Blood	W	222	3	4	275	30	6	255	8,491	ST-4821	5-3	10-2	F1-7	P1.5-3, 10-2: F1-7
Nm10	2017	Blood	B	222	3	58	275	386	18	255	5,664	ST-4821	20	23	F1-91	P1.20, 23: F1-91
Nm11	2017	Blood	B	222	3	58	275	386	18	255	5,664	ST-4821	20	23-1	F1-91	P1.20, 23-1: F1-91
Nm12	2017	Blood	B	222	273	58	275	30	18	8	15,886[Table-fn t1fns1]	ST-4821	20-1	23	F5-8	P1.20-1, 23: F5-8
Nm13	2017	Blood	C	222	3	58	275	30	5	255	4,821	ST-4821	21-2	9	F3-3	P1.21-2, 9: F3-3
Nm14	2018	Blood	B	222	231	406	26	6	2	16	15,887[Table-fn t1fns1]	–	19	13-13	F5-15	P1.19, 13-13: F5-15
Nm15	2018	Blood	B	222	3	58	275	30	5	255	4,821	ST-4821	7-2	14	F3-3	P1.7-2, 14: F3-3
Nm18	2019	Blood	B	222	3	58	275	386	18	255	5,664	ST-4821	20	23	F1-91	P1.20, 23: F1-91
Nm19	2019	Blood	B	222	3	58	275	386	18	255	5,664	ST-4821	20	23-3	F1-91	P1.20, 23-3: F1-91
Nm20	2019	CSF	B	222	3	58	275	386	18	255	5,664	ST-4821	20	23-19	F1-91	P1.20, 23-19: F1-91
Nm21	2019	CSF	B	46	11	88	635	22	6	410	12,301	–	18-25	9-18	F5-8	P1.18-25, 9-18: F5-8
Nm31	2019	Blood	Y	12	5	18	9	11	9	17	1,655	ST-23	5-1	10-1	F4-1	P1.5-1, 10-1: F4-1
Nm34	2021	Blood	Y	12	5	18	9	11	9	17	1,655	ST-23	5-1	10-1	F4-1	P1.5-1, 10-1: F4-1
Nm35	2021	CSF	B	222	231	406	55	6	2	16	5,666	ST-5666	19	13-13	F3-16	P1.19, 13-13: F3-16
**Carriage isolates**																
Nm16	2019	Throat swab	B	511	3	13	17	279	9	530	15,881[Table-fn t1fns1]	–	22	23-6	F5-7	P1.22, 23-6: F5-7
Nm17	2019	Throat swab	B	222	3	58	275	386	18	255	5,664	ST-4821	20	23-28	F1-91	P1.20, 23-28: F1-91
Nm22	2019	Throat swab	B	3	3	72	53	392	644	16	15,882[Table-fn t1fns1]	–	22	14	F4-46	P1.22, 14: F4-46
Nm23	2019	Throat swab	B	222	903[Table-fn t1fns1]	406	55	6	2	16	15,883[Table-fn t1fns1]	–	19	13-70	F5-15	P1.19, 13-70: F5-15
Nm24	2019	Throat swab	B	222	3	58	275	386	18	255	5,664	ST-4821	20	23	F1-91	P1.20, 23: F1-91
Nm25	2019	Throat swab	B	222	3	58	482	386	18	77	7,962	–	12-1	13-2	F4-21	P1.12-1, 113-2: F4-21
Nm26	2019	Throat swab	B	222	3	58	482	386	18	77	7,962	–	12-1	13-1	F4-21	P1.12-1, 113-1: F4-21
Nm27	2019	Throat swab	B	222	3	58	482	386	18	77	7,962	–	12-1	13-2	F4-21	P1.12-1, 113-2: F4-21
Nm28	2019	Throat swab	Un	46	11	88	24	22	1146[Table-fn t1fns1]	410	15,884[Table-fn t1fns1]	–	18-25	9-32[Table-fn t1fns1]	F5-8	P1.18-25, 9-32: F5-8
Nm29	2019	Throat swab	Un	222	3	58	275	386	18	255	5,664	ST-4821	20	23-3	F1-91	P1.20, 23-3: F1-91
Nm30	2019	Throat swab	Y	6	7	4	56	26	18	8	175	ST-175	5-1	2-2	F5-8	P1.5-1, 2-2: F5-8
Nm32	2020	Throat swab	B	1151[Table-fn t1fns1]	3	58	275	386	18	255	15,888[Table-fn t1fns1]	ST-4821	20	23-7	F1-91	P1.20, 23-7: F1-91
Nm33	2020	Throat swab	B	222	3	58	275	30	5	255	4,821	ST-4821	20	23-3	F3-3	P1.20, 23-3: F3-3

*^

^Meant new designed STs or alleles; Un meant untypable.*

Four MenW isolates were detected from MD cases. Three were grouped into CC ST-11, and one belonged to CC ST-4821. The hyperinvasive MenW CC ST-11-caused cases, epidemics, or even pandemics have emerged around the world. In 2000, a Hajj-linked MenW CC ST-11 outbreak swept the Middle East, the meningitis belt of Sub-Saharan Africa and South Africa (Mustapha et al., 2016). In recent years, endemic MenW CC ST-11 disease increased in South America, North America, Europe, and China (Zhou et al., 2013; Tsang et al., 2017; Eriksson et al., 2018; Rubilar et al., 2018). It was worth noting that two MenY CC ST-23 sporadic cases occurred in Taizhou and Haining in 2019 and 2021, respectively. All associated isolates belonged to ST1655 (Table 1). It has been known that most of the MenY MD was attributed to CC ST-23 (Hedberg et al., 2011; Toros et al., 2014). In Sweden, 62% of serogroup Y isolates from MD cases were CC ST-23 (Toros et al., 2014). During 1989–1991, approximately 2% of MD strains belonged to serogroup Y in the United States, whereas, by the mid-1990s, the percentage rose up to more than 30%. The increased proportion of MenY among all MD was attributed to CC ST-23 (Jackson and Wenger, 1993; Rosenstein et al., 1999; Harrison et al., 2006). Until early 2019, there were no MenY CC ST-23 isolates reported in China. MenY isolates in China were mostly associated with healthy carriers and grouped into three CCs, CC ST-175, CC ST-92, and CC ST-198 (Zhu et al., 2015; Wang et al., 2017; Shan et al., 2018). In March 2019, MenY CC ST-23–caused MD case first emerged in Guangdong Province, China (Li B. et al., 2019). MenY CC ST-23 MD cases increased gradually in China.

*porA* and *fetA* are two antigen encoding genes useful for meningococcal typing. PorA is a porin that exists on the outer membrane of most meningococcal isolates and contains two variable regions VR1 and VR2. FetA is an iron-regulated outer membrane protein with one variable region (Boan et al., 2014). We found predominant PorA subtypes P1.20, 23 [*n* = 3 (8.57%)], P1.20, 23-3 [*n* = 3 (8.57%)], P1.5, 2 [*n* = 3 (8.57%)], and P1.5-1, 10-1 [*n* = 3 (8.57%)]. One newly designated *porA* VR2 variant was P1.9-32, the peptide sequence was YVDEQGNYHA. The most prevalent FetA subtypes were F1-91 [*n* = 10 (28.57%)] and F5-8 [*n* = 4 (11.43%)]. High genotypic diversity was present in *porA* with DI of 0.9762. Meanwhile, the DI for *fetA* was 0.8968.

### Genome Sequencing-Based Phylogenetic Analysis of MenW CC ST-11 Strains

**FIGURE 3:**
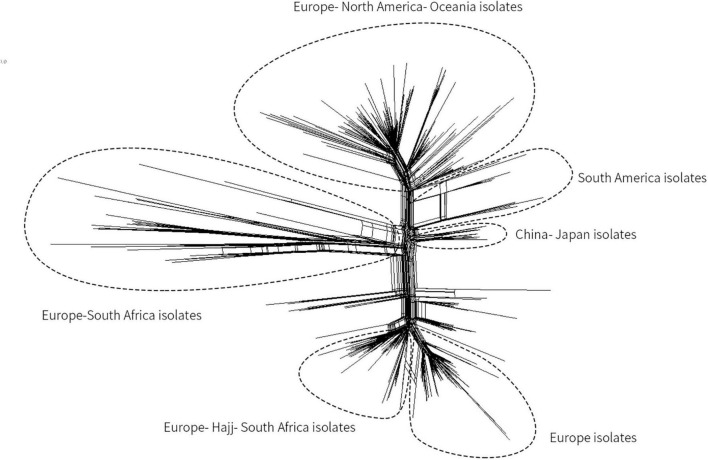
Core genome multilocus sequence typing (MLST) neighbor-net phylogenetic tree for the distribution of MenW CC ST-11 isolates from China and additional international isolates. The tree was constructed with SplitsTree4 version 4.13.1 (http://www.splitstree.org).

Since a Mecca Saudi Arabia Hajj–associated outbreak in 2000, Hyperinvasive MenW CC ST-11 has swept the globe (Ladhani et al., 2015; Mustapha et al., 2016). In China, before 2011, only three cases of MenW MD were reported (Shao et al., 2010), whereas from February 2011 to June 2012, MD caused by MenW increased (11 cases total). All associated isolates were identified as CC ST-11 (Zhou et al., 2013). Herein, based on genome sequencing, the phylogenetic relationship of MenW CC ST-11 isolates from China with international isolates from Saudi Arabia Hajj-associated outbreak, African meningitis belt, South America, North America, South Africa, Europe, and Oceania was analyzed to speculate the origin of Chinese isolates. Phylogeny results indicated that all Chinese isolates (*N* = 9, three were from this study) were genetically linked and grouped together with isolates from Japan, separated from other MenW CC ST-11 isolates including the Europe–Hajj–South Africa branch, Europe branch, Europe–South Africa branch, Europe–North America–Oceania branch, and South America branch (Figure 3). Previous epidemiological studies (Mustapha et al., 2016) demonstrated that following a Mecca Saudi Arabia outbreak in 2000, a majority of globally spread MenW CC ST-11 evolved from the Hajj clone. Until recent years, significant genetic heterogeneity among MenW CC ST-11 strains occurred. While the Hajj clone continuously spread in the Middle East, South Africa had cocirculated with the Hajj clone and other MenW CC ST-11 strains. According to the phylogenetic analysis in our study, cocirculation of genetically distinct MenW CC ST-11 isolates occurred extensively in the African meningitis belt, Europe, and South Africa. MenW CC ST-11 isolates from East Asia and South America might have evolved locally.

### Antibiotic Susceptibility of *N. meningiti*dis Isolates

The susceptibilities of *Nm* isolates to 12 antibiotics are shown in Table 4. All 35 strains were susceptible to minocycline, ceftriaxone, cefotaxime, meropenem, and chloramphenicol; 74.29% (26/35) of strains were susceptible to azithromycin. The number of intersusceptible and resistant strains to azithromycin could not be identified because of the lack of associated breakpoints. The MIC of the undetermined strains ranged from 1 to 12 μg/mL. Resistance to rifampin, ciprofloxacin, penicillin, trimethoprim-sulfamethoxazole, and levofloxacin was presented by 1 isolate (2.86%), 32 isolates (91.43%), 8 isolates (22.86%), 30 isolates (85.71%), and 32 isolates (91.43%), respectively. In the empirical MD therapy scheme, the third-generation cephalosporins (e.g., ceftriaxone and cefotaxime) were recommended as the treatment of choice. Our results and previous reports (Gorla et al., 2018a) showed that *Nm* isolates with different sources had different cephalosporin susceptibility frequency, such as ranging from 100 to 64.2% for cefotaxime. Penicillin also remains one choice for MD treatment, although the increased resistance of *Nm* isolates to this antibiotic from different countries has been reported (Harcourt et al., 2015). To date, the resistance profiles to penicillin of *Nm* isolates in China were still ambiguous. According to the results herein, a relatively high level of penicillin resistance was observed. Fluoroquinolones, mainly including ciprofloxacin, levofloxacin, and norfloxacin, have been used for MD prophylaxis for high-risk adult close contacts to prevent secondary MD cases and outbreaks (Chen et al., 2015; Gorla et al., 2018b). Compared with previously reported resistance rates (0.0–84%) to ciprofloxacin of *Nm* isolated from different regions of different countries (Chen et al., 2015; Gorla et al., 2018b; Tefera et al., 2020), a consistent resistance rate was observed among our isolates to that from Shanghai, China, since 2005. *Nm* isolates from China presented a significantly higher ciprofloxacin resistance level than that from Brazil, South Korea, and so on. Among the ciprofloxacin-resistant isolates, 62.50% (20/32) were assigned either CC ST-4821 (*n* = 17) or CC ST-11 (*n* = 3), including eight (22.86%) multidrug-resistant strains, resistant to ciprofloxacin/penicillin/trimethoprim-sulfamethoxazole/levofloxacin and rifampin/ciprofloxacin/penicillin/trimethoprim-sulfamethoxazole/levofloxacin, respectively.

**TABLE 4:** Antibiotic susceptibility of *Nm* isolates from both MD cases and carriage.

Antimicrobial class	Antimicrobial agents	MIC (μg/mL) interpretive criteria			No. of isolates (%)		
		Susceptible	Intermediate	Resistant	Susceptible	Intermediate	Resistant
Penicillins and β-lactam/β-lactamase inhibitor combinants	Penicillin	≤0.06	0.12–0.25	≥0.5	17 (48.57%)	10 (28.57%)	8 (22.86%)
	Ampicillin	≤0.12	0.25–1	≥2	23 (65.71%)	12 (34.29%)	0 (0%)
	Ceftriaxone	≤0.12	–	–	35 (100%)	–	–
	Cefotaxime	≤0.12	–	–	35 (100%)	–	–
Phencols	Chloramphenicol	≤2	4	≥8	35 (100%)	0 (0%)	0 (0%)
Ansamycins	Rifampin	≤0.5	1	≥2	34 (97.14%)	0 (0%)	1 (2.86%)
Quinolones	Levofloxacin	≤0.03	0.06	≥0.12	3 (8.57%)	0 (0%)	32 (91.43%)
	Ciprofloxacin	≤0.03	0.06	≥0.12	3 (8.57%)	0 (0%)	32 (91.43%)
Macrolide	Azithromycin	≤2	–	–	26 (74.29%)	–	–
Tetracyclines	Minocycline	≤2	–	–	35 (100%)	–	–
Folate metabolic pathway inhibitor	Sulfamethoxazole-trimethoprim	≤0.12/2.4	0.25/4.75	≥0.5/9.5	5 (14.29%)	0 (0%)	30 (85.71%)
Carbapenems	Meropenem	≤0.25	–	–	35 (100%)	0 (0%)	0 (0%)

## Conclusion

In summary, a comprehensive study of epidemiology of MD, healthy carriage, and characteristics of *Nm* isolates in Zhejiang Province, China, was performed, providing valuable data for understanding the epidemiological characteristics of MD and carriage, as well as the need for and impact of vaccination. During the study period, the annual incidence of MD remained relatively low, ranging from 0.0017 to 0.0183 per 100,000 population. The highest age-specific incidence was in the group younger than 1 year. MenB presented predominantly in both MD cases and healthy population; a significant increase in MenB incidence was also observed from 2013 to 2019. Combined with previously published data, the dominant proportion and increasing incidence of Men B should cause more concern in China. It was worth noting that two MenY CC ST-23 cases occurred in 2019 and 2021. MenY CC ST-23 MD cases increased gradually in China. To the best of our knowledge, this is the first time that the phylogenetic relationship of MenW CC ST-11 isolates from China with international isolates was analyzed to speculate the origin of Chinese isolates based on genome sequencing. In addition, the relatively high resistance rate (22.86%) to penicillin among *Nm* isolates and the discovery of multidrug-resistant strains indicate a potential public health problem.

## Data Availability Statement

The datasets presented in this study can be found in online repositories. The names of the repository/repositories and accession number(s) can be found below: https://pubmlst.org/bigsdb?db=pubmlst_neisseria_isolates, 105802–105807.

## Author Contributions

YZ and XD: experimental operation, data processing, and manuscript writing. YJ: genome sequencing and data processing. JZ, LZ, LM, and HL: experimental operation. PY and HH: manuscript writing and revision. All authors contributed to the article and approved the submitted version.

## Conflict of Interest

The authors declare that the research was conducted in the absence of any commercial or financial relationships that could be construed as a potential conflict of interest.

## Publisher’s Note

All claims expressed in this article are solely those of the authors and do not necessarily represent those of their affiliated organizations, or those of the publisher, the editors and the reviewers. Any product that may be evaluated in this article, or claim that may be made by its manufacturer, is not guaranteed or endorsed by the publisher.

## Footnotes

1
https://pubmlst.org/neisseria/

2
http://www.splitstree.org
